# Comparing fusion and complication rates after instrumented versus uninstrumented fusion for lumbar spondylolisthesis: A systematic review and meta analysis of randomized controlled trials with trial sequential analysis

**DOI:** 10.1016/j.jor.2025.05.059

**Published:** 2025-05-27

**Authors:** Omkar S. Anaspure, Pierce Davis, Anthony N. Baumann, Meredith Kossoff, Gordon Preston, Keegan T. Conry, Jacob C. Hoffmann

**Affiliations:** a Perelman School of Medicine, University of Pennsylvania, Philadelphia, PA, USA; b Department of Rehabilitation Services, University Hospitals, Cleveland, OH, USA; c Department of Orthopedic Surgery, Cleveland Clinic Akron General, Akron, OH, USA

**Keywords:** Lumbar spine, Degenerative spondylolisthesis, Lumbar spinal fusion, Instrumented, Uninstrumented

## Abstract

**Introduction:**

Spinal fusion is a common treatment for degenerative or isthmic lumbar spondylolisthesis (LS) in adult patients, where vertebral slippage can lead to significant neurological impairment. However, debate exists regarding the exact fixation method, as fusion and complication rates may differ via instrumented fusion (IF) or uninstrumented fusion (UIF). Therefore, the purpose of this study is to investigate the high-level literature for the fusion and complication rates associated with IF versus UIF to guide decision-making.

**Methods:**

This systematic review and meta-analysis utilized PubMed, SCOPUS, and Web of Science through September 15th, 2024, to assess the fusion and complication rates associated with IF versus UIF for LS. Inclusion criteria included randomized controlled trials (RCTs) only. The primary outcomes were rates of fusion, reoperation, and complication rates. Statistical analysis included relative risk (RR) with 95 % confidence intervals (CI) along with trial sequential analysis (TSA) and assessment of fragility index (FI).

**Results:**

A total of five RCTs were included in this study out of the 799 articles initially retrieved. Included patients (n = 286; 72.02 % female) had a mean age of 60.97 years and underwent either IF (n = 150) or UIF (n = 136) for LS with a mean follow-up of 2.31 years. Roughly 26.92 % of patients had isthmic LS (n = 77) and 73.08 % of patients had degenerative LS (n = 209) with 98.91 % of patients (n = 182/184) having a grade 1 or 2 LS. Patients who underwent IF had a statistically significant higher rate of fusion as compared to patients who underwent UIF for LS (90.7 % versus 48.5 %; RR: 1.96; 95 % CI: [1.23, 3.13]; p = 0.005) with robust evidence (FI: 11 patients). However, there was no statistically significant difference in reoperation rates (10.4 % versus 2.7 %; RR: 1.05; 95 % CI: [0.97,1.13]; p = 0.264) or total complication rates (7.3 % versus 2.4 %; RR: 0.90; 95 % CI: [0.90, 1.02]; p = 0.228) between patients who underwent IF versus UIF for LS. TSA for all primary outcomes demonstrated a Z-curve that did not cross the required information size, suggesting more research is needed for definitive conclusions on this topic. Qualitatively, two RCTs reported greater operative time (OT) and estimated blood loss (EBL) for IF as compared to UIF for LS.

**Conclusion:**

Among adult patients with LS, IF resulted in a robust and statistically significant higher fusion rate as compared to UIF, although more research is needed for definitive conclusions. However, there was no statistically significant difference in reoperation or total complication rates for IF versus UIF for LS.

## 1 Introduction

Spinal fusion is a standard treatment for isthmic and degenerative lumbar spondylolisthesis (LS), a condition where one vertebra slips over another, potentially causing pain and neurological symptoms.^1^, ^2^, ^3^ Fusion stabilizes the affected vertebrae and prevents slippage, which can alleviate symptoms and improve functional outcomes.^1^^,^^4^^,^^5^ Pedicle screws are often chosen in cases of instrumented fusion (IF) where additional support is deemed necessary, as opposed to fusion without pedicle screws. In uninstrumented fusion (UIF), fusion relies on bone graft packed along the posterolateral aspect of the spine between the transverse processes of the vertebrae to stimulate fusion between adjacent vertebra.^2^^,^^3^^,^^6^, ^7^, ^8^, ^9^, ^10^ Instrumentation may offer better alignment and enhanced fusion rates, particularly in patients with primary degenerative instability, where IF has shown superior outcomes compared to UIF.^8^, ^9^, ^10^ However, the use of pedicle screws in IF may ultimately lead to increased complications, including increased reoperation rates due to screw malpositioning, pseudarthrosis, or hardware removal.^3^^,^^6^^,^^11^

Although lumbar pedicle screws offer favorable outcomes in certain subgroups, the question remains: Is the added stability worth the potential complications for LS? While randomized controlled trials (RCTs) are the gold standard for comparing interventions, there is a paucity of RCTs that specifically address the use of pedicle screw in fusion procedures for LS. A recent systematic review by Hirase et al. (2022) attempted to address this disparity, but their study was limited to three RCTs, preventing meta-analysis and relying on the narrative synthesis of a small number of heterogeneous, nonrandomized studies.^12^ Although their study begins to provide insight into the adequate bony arthrodesis capabilities of IF in LS, more RCTs have been published in the literature in the interim. Additionally, Jia et al. (2024) conducted a network meta-analysis comparing interventions for degenerative LS, but lacked data on IF versus UIF, further underscoring the need for a more comprehensive study focused on this critical comparison.^13^ Furthermore, the recent commentary by Forte et al. (2023) on the recent RCT by Andresen et al. (2023) emphasized the importance of meta-analysis in overcoming the limitations of small trials and endorsed the use of the Fragility Index (FI) to assess the reliability of binary outcomes such as reoperation rates, emphasizing the need for stronger, more reliable findings while giving direction for future research.^14^

Prior to recommending the use of pedicle screw fixation during fusion procedures for LS, it is prudent to evaluate the robustness and conclusiveness of the current RCT evidence existing in the current literature. At this time, the question remains whether the high-level evidence from existing RCTs can sufficiently guide clinical decisions on lumbar fusion constructs when treating patients with LS. Therefore, the purpose of this systematic review and meta-analysis is to evaluate the rates of fusion, reoperation, and complications following IF versus UIF for patients with LS, incorporating trial sequential analysis (TSA) and FIs to assess the strength of the evidence and guide surgeon decision making in the interim.^15^, ^16^, ^17^, ^18^

## 2 Methods

### 2.1 Study creation and initial search

This study is a systematic review and meta-analysis examining rates of fusion and complications in patients with LS undergoing either IF or UIF by querying PubMed, SCOPUS, and Web of Science from database inception until September 15th, 2024. Search terms for each database were (fusion OR “instrumented fusion”) AND (lumbar) AND (spondylolisthesis) AND (random). This study was performed under the recent Preferred Reporting Items for Systematic Reviews and Meta-Analyses (PRIMSA) guidelines. This study was preregistered on the Open Science Framework (osf.io/8k7u4).

### 2.2 Inclusion and exclusion criteria

Inclusion criteria were RCTs that compared adult patients (older than 18 years old) with LS who underwent IF versus UIF and reported clinical outcomes of patients after fusion surgery. Exclusion criteria were observational studies (retrospective studies, prospective cohort studies without randomization, case series, and case reports), articles without full-text, articles not in English, studies not involving LS, outcomes not specific to LS, and studies that failed to report any clinical outcomes.

### 2.3 Article screening process

After the search algorithm was executed in each of the four databases for the initial search, all articles were uploaded into Rayyan, a public website used for systematic reviews.^19^ Two screeners performed a manual de-duplication of articles, and subsequently performed article screening based on title and abstract. This was followed by full-text screening based on inclusion and exclusion criteria to obtain the final article count. Any conflicts during the article screening process were resolved by the first author.

### 2.4 Data extraction

Data extraction was completed by two authors. Data extracted included first author, year of publication, procedure type, number of patients, sex, average age, type of LS, grade of LS, follow-up time, operative time, fusion outcomes, reoperations, complications, and any other relevant qualitative data for LS.

### 2.5 Primary outcome measures

The primary outcome measures assessed in this study by meta-analysis were postoperative bony fusion success assessed at final follow-up, number of reoperations experienced, and total complications experienced at final follow-up. Secondary outcomes included operative time and estimated blood loss.

#### 2.5.1 Article quality grading

The bias of the included RCTs was graded using the Cochrane Collaboration's tool for assessing RCTs.^20^ The Cochrane Collaboration's tool for assessing RCTs uses seven areas of bias that were graded as “high risk,” “low risk” or “unclear”: random sequence generation, allocation concealment, blinding of participants and personnel, blinding of outcome assessment, attrition bias, selective bias, and other bias.^20^ The existence of publication bias was not assessed by funnel plots because of few articles included in this study, although a large range of publication dates were found.^21^^,^^22^ The Grading of Recommendations Assessment, Development and Evaluation (GRADE) score were calculated for individual article quality assessment and reported as a scale from very low, low, moderate, and high.^23^

### 2.6 Statistical analysis

This study utilized the Statistical Package for the Social Sciences (SPSS) version 29.0 (Armonk, NY: IBM Corp) for statistical analysis. Frequency weighted means and other descriptive statistics were used to describe the data where no statistical significance could be calculated. A random-effects binary outcomes meta-analysis was performed using relative risk (RR) and 95 % confidence intervals (CIs) for effect size for statistical comparison between studies with binary outcomes. A forest plot was generated to depict the relationship between variables where able. To address the issue of having a limited number of available trials, we performed the TSA utilizing software from the Copenhagen Trial Unit.^17^ Data was collected and TSA was conducted using the following parameters: α = 5 %, power = 80 %, and observed relative risk reduction of 20 %, adjusted for heterogeneity from the meta-analysis. Using the O'Brien-Fleming α-spending function, we applied cumulative Z-curve analysis to monitor the accumulating data. The FI is determined by converting one patient in the group (control or experimental group) from a “non-event” to an “event” outcome and recalculating a two-sided Fisher's exact test until the p value meets or exceeds 0.05.^24^^,^^25^ Outcomes were deemed robust if the FI was greater than the number of patients lost to follow-up/drop-out, and fragile if the number of patients lost to follow-up/drop-out was greater than FI.

## 3 Results

### 3.1 Initial search results

**Fig. 1:**
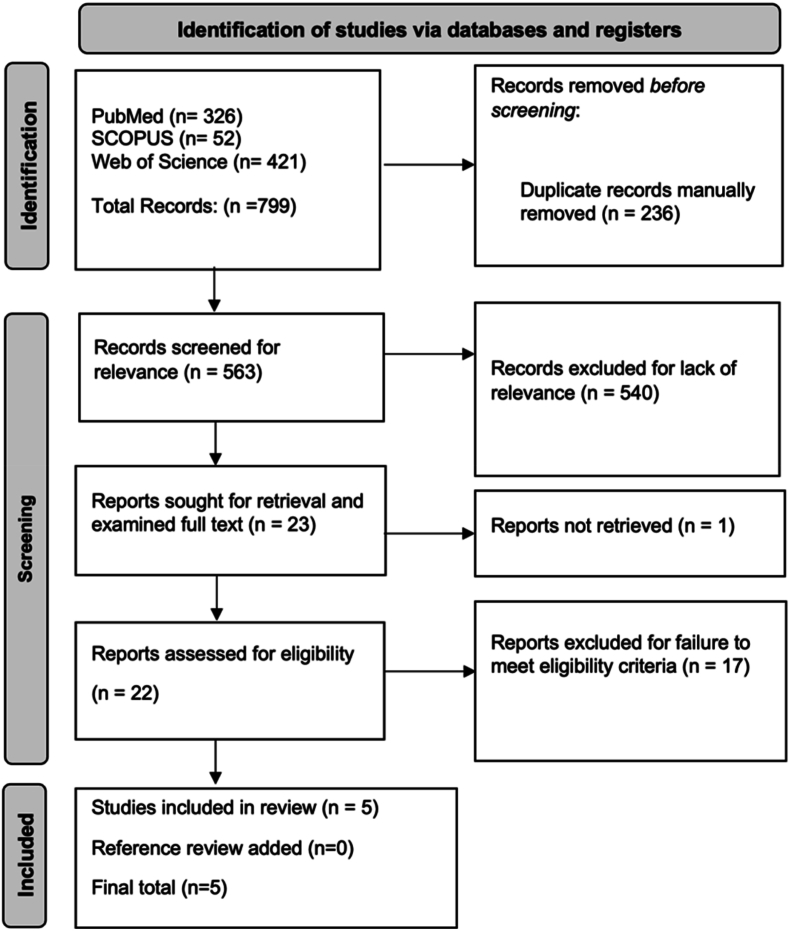
The Preferred Reporting Items for Systematic Reviews and Meta-Analyses (PRISMA) diagram outlining the entire search progress, from initial search in four databases to final article inclusion.

The database search resulted in 799 articles; after automated de-duplication, 563 articles remained. After title and abstract screening, 23 articles were included for the full-text analysis. After this process, five RCTs met inclusion criteria and were included in the data extraction process (Fig. 1).^7^^,^^11^^,^^26^, ^27^, ^28^

Article Quality Results.

**Fig. 2:**
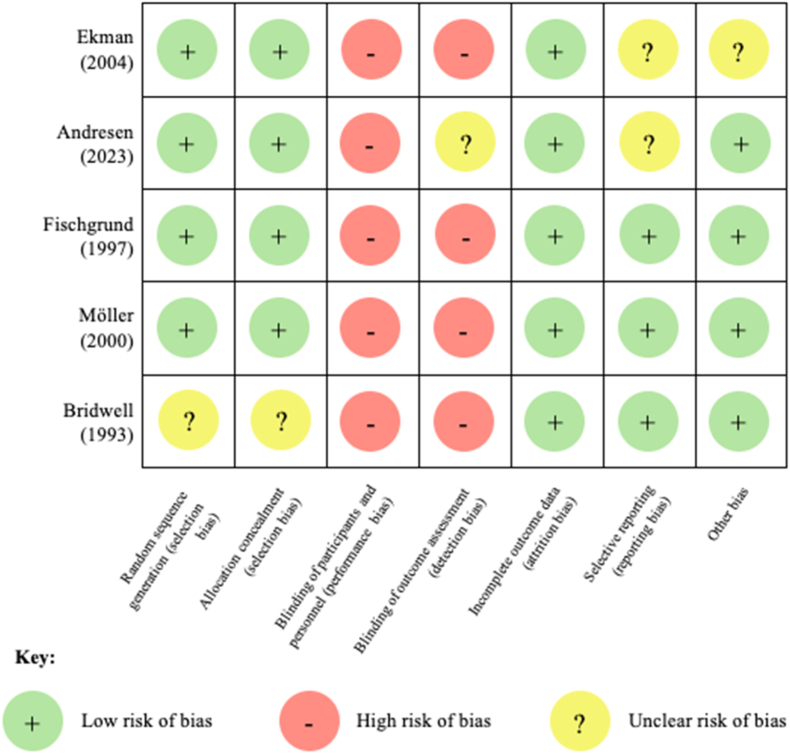
Outcomes of the Cochrane Risk of Bias 2.0 tool for randomized trials; n = 5 trials.

Of the five included articles, all were RCTs per our inclusion criteria. All RCTs had low risk of bias in regard to random sequence generation, allocation concealment, and attrition bias. Due to the surgical nature of the intervention, no study was able to prevent performance or outcome bias (Fig. 2). The study by Ekman et al. (2004) served as a followup to the initial study done by Moller et al. (2000), thus the cohorts in both studies are identical. However, only Moller et al. (2000) reported clinical outcomes relevant to the current study, and as such, only those values were included in pooled statistics. Due to this, there are four unique studies included in the current meta anlysis.

### 3.2 Patient and study characteristics

Across the four unique RCTs, there was 286 adult patients with LS included in this study. Of these 286 patients, patients with IF comprised of 52.44 % (n = 150) of the cohort whereas patients with UIF comprised of 47.55 % (n = 136) of the cohort (Table 1). For specifics related to LS, 26.92 % (n = 77) of the included patients had isthmic LS and 73.08 % (n = 209) of the included patients had degenerative LS. Additionally, 98.91 % (n = 182/184) of the patients had a slip grade of either 1 or 2, suggesting low grade LS. The FWM age of the cohort was 60.97 ± 13.57 years and a total of 80 (27.97 %) males and 206 (72.02 %) females were included. The mean follow-up time for the entire patient cohort was 2.31 ± 0.45 years.

**Table 1:** Study and patient demographic information including surgery type, mean age, gender, slip grade, follow-up time, and level impacted.

First Author (year)	Surgery Type	Groups	# of patients	Age	Gender	Follow-up Length	LS Type	Slip Grade	Level Impacted
Ekman (2004)	Posterolateral fusion	Uninstrumented fusion	40	39	Male (n = 22) Female (n = 18)	9 years	Isthmic spondylolisthesis	Grade 1 (n = 27, 68 %), Grade 2 (n = 12, 30 %), Grade 3 (n = 1, 3 %)	L5 (n = 33, 83 %), L4 (n = 6, 15 %), L4-L5 (n = 1, 3 %)
		Instrumented fusion	37	39	Male (n = 16) Female (n = 21)			Grade 1 (n = 20, 54 %), Grade 2 (n = 16, 43 %), Grade 3 (n = 1, 3 %)	L5 (n = 31, 834), L4 (n = 5, 14 %), L4-L5 (n = 1, 3 %)
Andresen (2023)	Posterolateral fusion	Uninstrumented fusion	53	72.3 (70.5–74.1)	Male (n = 5) Female (n = 48)	2 years	Degenerative spondylolisthesis	Grade 1 or 2	L3-L4 (n = 4, 7.5 %), L4-L5 (n = 47, 88.7 %), L5-S1 (n = 2, 3.8 %)
		Instrumented fusion	54	70.8 (69.1–72.4)	Male (n = 11) Female (n = 43)				L3-L4 (n = 5, 9.3 %), L4-L5 (n = 48, 88.9 %), L5-S1 (n = 1, 1.9 %)
Fischgrund (1997)	Posterolateral fusion after decompression	Uninstrumented fusion	33	66 (52–80)	Male (n = 6) Female (n = 27)	28 months	Degenerative spondylolisthesis	–	–
		Instrumented fusion	35	69 (53–86)	Male (n = 7) Female (n = 28)			–	–
Möller (2000)	Posterolateral fusion	Uninstrumented fusion	40	39	Male (n = 22) Female (n = 18)		Isthmic spondylolisthesis	Grade 1 (n = 27, 68 %), Grade 2 (n = 12, 30 %), Grade 3 (n = 1, 3 %)	L5 n = 33 (83 %), L4 (n = 6, 15 %), L4-L5 (n = 1, 3 %)
		Instrumented fusion	37	39	Male (n = 21) Female (n = 18)	–		Grade 1 (n = 20, 54 %), Grade 2 (n = 16, 43 %), Grade 3 (n = 1, 3 %)	L5 n = 31 (84 %), L4 (n = 5, 14 %), L4-L5 (n = 1, 3 %)
Bridwell (1993)	Posterior fusion with autogenous iliac crest bone graft	Uninstrumented fusion	10	65	Male (n = 4) Female (n = 6)	3 years, 9 months	Degenerative spondylolisthesis	–	L3-L4 (n = 1, 11.1 %), L4-L5 (n = 8, 88.9 %)
		Instrumented fusion	24	64	Male (n = 4) Female (n = 20)	3 years		–	L3-L4 (n = 4, 16.7 %), L4-L5 (n = 20, 83.3 %)

### 3.3 Fusion outcomes after instrumented or uninstrumented posterolateral spinal fusion

**Fig. 3:**
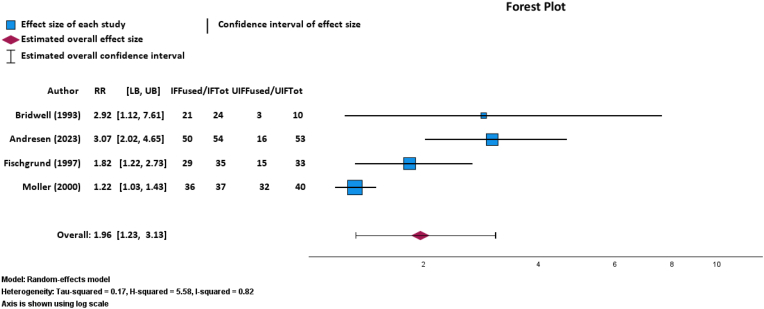
Random-effects model forest plot demonstrating relationship between successful bony fusion and instrumented or uninstrumented fusion for four RCTs. Effect measures used were RRs and CIs. Abbreviations: IFFused, number of patients with instrumented fusion who achieved successful bony fusion; IFTot, total number of patients who received instrumented fusion; UIFFused, number of patients with uninstrumented fusion who achieved successful bony fusion; UIFTot, total number of patients who received uninstrumented fusion; RR, risk ratio; CI, confidence interval. Heterogeneity: Tau-squared = 0.17, H-squared = 5.58, I-squared = 0.82. Axis is shown using log scale.

**Fig. 4:**
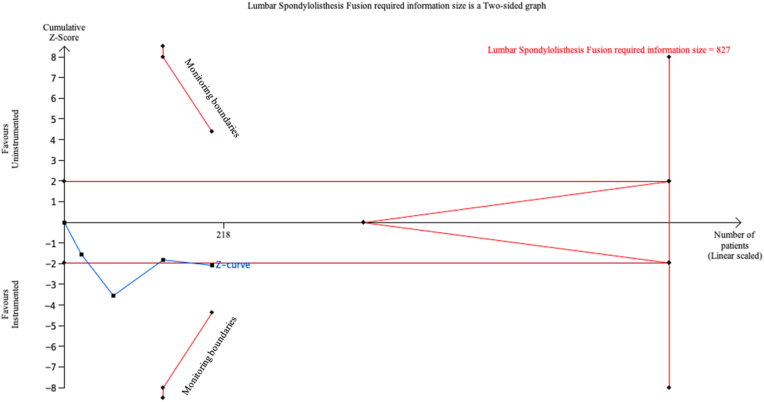
Trial Sequential Analysis (TSA) comparing fusion outcomes between instrumented fusion (IF) and uninstrumented fusion (UIF) for lumbar spondylolisthesis (LS) using a two-sided alpha of 5 %, 80 % power, and a relative risk reduction (RRR) of 20 %, adjusted for heterogeneity. A required information size of 827 patients was calculated, with 202 patients included in the analysis. The cumulative Z-curve (blue line) did not cross the required information size boundary (red line), indicating that the current evidence is inconclusive.

There was a statistically significant increase in fusion after IF (n = 136 out of 150 cases; 90.67 % fused spines) as compared to UIF (n = 66 out of 136 cases; 48.53 % fused spines) via a random-effects meta-analysis of four RCTs (p = 0.005; RR: 1.96; 95 % CI, 1.23–3.13; Fig. 3).^7^^,^^11^^,^^26^^,^^28^This finding was robust, with an FI of 11 patients needed to alter the statistical significance of fusion outcomes in patients undergoing IF versus UIF. The GRADE certainty of this outcome was moderate. TSA of fusion outcomes after IF or UIF determined a required information size of 827 patients. At the current number of 202 fusions, the cumulative Z-curve did not cross the monitoring boundary (p = 0.03) or the required information size threshold, indicating that further trials are needed to reach a conclusive result (Fig. 4).

### 3.4 Reoperations after instrumented or uninstrumented posterolateral spinal fusion

**Fig. 5:**
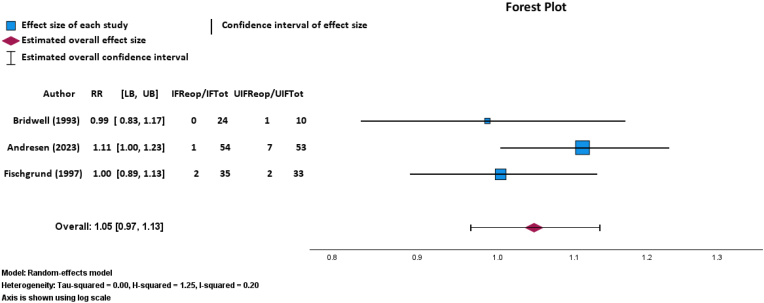
Random-effects model forest plot demonstrating relationship between number of reoperations and instrumented or uninstrumented fusion for three RCTs. Effect measures used were RRs and CIs. Abbreviations: IFReop, number of patients with instrumented fusion who needed reoperation; IFTot, total number of patients who received instrumented fusion; UIFReop, number of patients with uninstrumented fusion who needed reoperation; UIFTot, total number of patients who received uninstrumented fusion; RR, risk ratio; CI, confidence interval. Heterogeneity: Tau-squared = 0.00, H-squared = 1.25, I-squared = 0.20. Axis is shown using log scale.

**Fig. 6:**
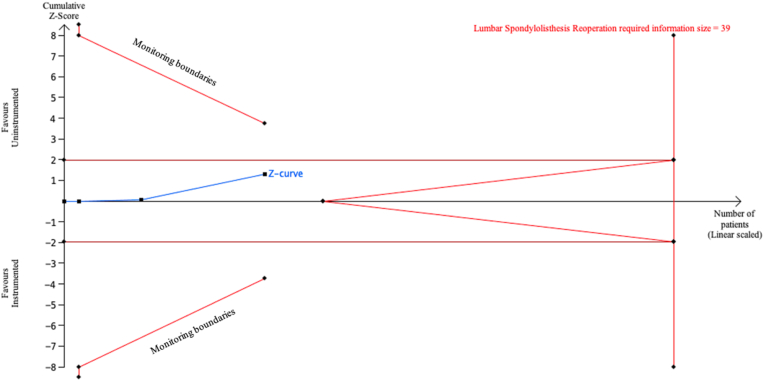
Trial Sequential Analysis (TSA) comparing reoperation between instrumented fusion (IF) and uninstrumented fusion (UIF) for lumbar spondylolisthesis (LS) using a two-sided alpha of 5 %, 80 % power, and a relative risk reduction (RRR) of 20 %, adjusted for heterogeneity. A required information size of 39 patients was calculated, with 13 patients included in the analysis. The cumulative Z-curve (blue line) did not cross the required information size boundary (red line), indicating that the current evidence is inconclusive.

There was no statistically significant difference in reoperation after IF (n = 3 out of 113 cases; 2.65 % required reoperations) versus UIF (n = 10 out of 96 cases; 10.42 % required reoperations) via a random-effects meta-analysis of three RCTs (p = 0.264; RR: 1.05; 95 % CI, 0.97–1.13; Fig. 5).^11^^,^^26^^,^^28^This finding was fragile, with an FI of 1 patient needed there to be a significant difference in the number of reoperations in patients. The GRADE certainty of this outcome was moderate. TSA of reoperation outcomes after IF or UIF determined a required information size of 39 patients. At the current number of 13 reoperations, the cumulative Z-curve did not cross the monitoring boundary (p = 0.199) or the required information size threshold, indicating that further trials are needed to reach a conclusive result (Fig. 6).

### 3.5 Complications after instrumented or uninstrumented posterolateral spinal fusion

**Fig. 7:**
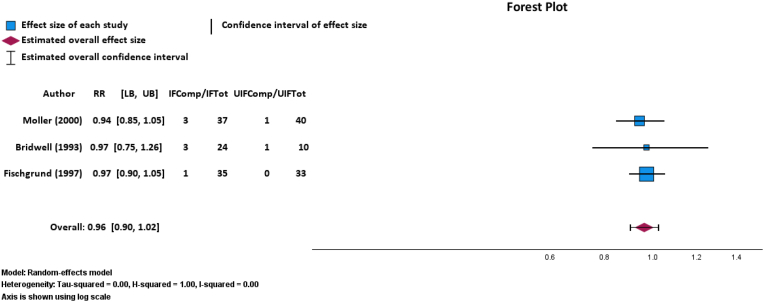
Random-effects model forest plot demonstrating relationship between total complications after and instrumented or uninstrumented fusion for three RCTs. Effect measures used were RRs and CIs. Abbreviations: IFComp, number of patients with instrumented fusion who experienced postoperative complication; IFTot, total number of patients who received instrumented fusion; UIFComp, number of patients with uninstrumented fusion who experienced postoperative complications; UIFTot, total number of patients who received uninstrumented fusion; RR, risk ratio; CI, confidence interval. Heterogeneity: Tau-squared = 0.00, H-squared = 1.00, I-squared = 0.00. Axis is shown using log scale.

**Fig. 8:**
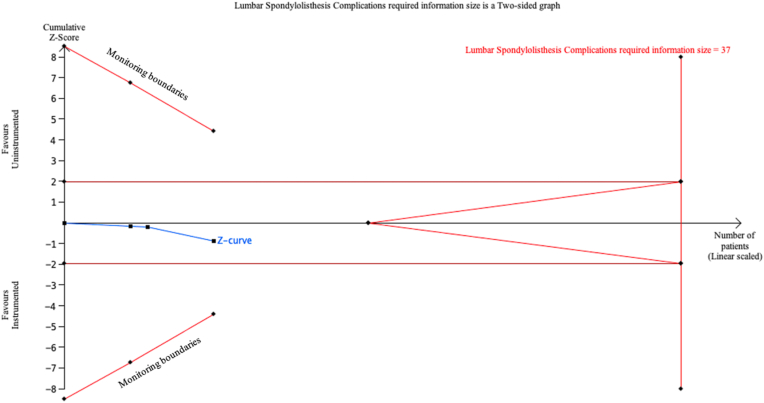
Trial Sequential Analysis (TSA) comparing complications between instrumented fusion (IF) and uninstrumented fusion (UIF) for lumbar spondylolisthesis (LS) using a two-sided alpha of 5 %, 80 % power, and a relative risk reduction (RRR) of 20 %, adjusted for heterogeneity. A required information size of 37 patients was calculated, with 9 patients included in the analysis. The cumulative Z-curve (blue line) did not cross the required information size boundary (red line), indicating that the current evidence is inconclusive.

There was no statistically significant difference in the number of complications after IF (n = 7 out of 96 cases; 7.29 % required reoperations) versus UIF (n = 2 out of 83 cases; 2.41 % required reoperations) via a random-effects meta-analysis of three RCTs (p = 0.228; RR: 0.90; 95 % CI, 0.90–1.02; Fig. 7).^7^^,^^26^^,^^28^ This finding was fragile, with an FI of 4 patients needed there to be a significant difference in fusion outcomes in patients. The GRADE certainty of this outcome was moderate. TSA of complications after IF or UIF determined a required information size of 39 patients. At the current number of 9 complications, the cumulative Z-curve did not cross the monitoring boundary (p = 0.377) or the required information size threshold (Fig. 8), indicating that further trials are needed to reach a conclusive result.

### 3.6 Operative time during instrumented or uninstrumented posterolateral spinal fusion

Moller et al. (2000) found that the mean operation time was significantly greater in the IF group (298 min; range, 170–450 min) compared to the UIF group (201 min; range, 85–470 min) (p < 0.0001).^7^ Similarly, Andresen et al. (2023) found that patients in the instrumented group had a longer operative time (123.4 min; range, 116.4–130.3 min) compared with the uninstrumented group (88.8 min; range, 82.3–95.2 min) (p < 0.001).^11^

### 3.7 Blood loss after instrumented or uninstrumented posterolateral spinal fusion

Moller et al. (2000) found that the intraoperative blood loss was significantly greater in the IF group (1517 mL; range, 360–7000 mL) compared to the UIF group (861 mL; range, 100–3100 mL) (p < 0.0001).^7^ Similarly, Andresen et al. (2023) found that patients in the IF group had greater perioperative and postoperative blood loss (373.5 ml; range, 326.8–420.1 ml) compared to patients in the UIF group (244.5 ml; range, 209.4–279.7 ml) (p < 0.001).^11^

## 4 Discussion

To our knowledge, this study represents the first systematic review and meta-analysis with TSA to compare postoperative outcomes of IF versus UIF for LS using exclusively high-level RCT data. Although previous reviews have addressed fusion techniques broadly, this study uniquely focuses on the specific impact of instrumentation in patients with LS. Our findings indicate that IF achieves robust and significantly higher fusion rates than UIF (90.7 % vs. 48.5 %), though this advantage did not translate to statistically significant differences between IF and UIF regarding reoperation or complication rates. Qualitative data from two studies suggest that IF may involve longer operative times and increased blood loss, though these findings are limited to a few numbers of studies with small cohort sizes. TSA results further underscore that the current evidence base remains inconclusive with future RCTs possibly changing the results. Furthermore, the FI analysis highlights the frailty of primary outcomes such as reoperation and complications. Additional RCTs are needed to substantiate observed qualitative trends in perioperative parameters and to provide additional context regarding the primary outcomes examined in this study.

The degree of instability in lumbar spondylolisthesis varies significantly across patients, ranging from mild anterolisthesis with minimal neural compression to severe, symptomatic instability requiring robust stabilization.^29^^,^^30^ Similarly, our study primarily comprised of patients of both grade 1 (0–25 % slip) and grade 2 (26–50 % slip) patients. Mild cases of instability may achieve adequate outcomes with UIF, but evidence is limited regarding if the level of fusion achieved with IF in cases of higher-grade slips or significant neural compression justifies the currently unclear risk profile associated with instrumentation. Instrumentation with pedicle screws can provide enhanced mechanical stability, leading to higher fusion rates, as seen in the meta-analysis by Ye et al. (2014), where IF showed a significantly higher fusion rate (77–97 %) compared to UIF (45–67 %) across studies.^31^ This increase in fusion success is seen without sacrificing postoperative clinical outcomes. For example, Wu et al.'s (2024) network meta-analysis found no statistically significant differences in ODI improvements between various fusion techniques, suggesting that the added stability of IF may not impact the magnitude of functional recovery.^5^ Similarly, Abdu et al. (2018), in the well-known SPORT trial, reported that while fusion rates were higher with IF, long-term functional outcomes and pain scores did not differ significantly between IF and UIF.^32^ Individual study trends by Fischgrund et al. (1997) and Andresen et al. (2023) agree with We et al. (2024) and Abdu et al. (2018), where fusion rates for IF were significantly higher than UIF (82.8 %–94 % vs. 31 %–45.4 %, respectively) but no significant differences in long-term functional outcomes like ODI or pain relief. Overall, our pooled study findings agree with that of Wu et al. (2024) and Abdu et al. (2018) findings, where IF shows significantly greater fusion rates compared to UIF with no significant differences in reoperation or complications.

Based on these findings, it's crucial to consider that the higher fusion rates achieved with IF may not necessarily translate into improved symptomatic relief, which is often the primary goal for patients undergoing fusion surgery. This must be balanced with the long-term construct stability which IF may be better suited to provide for patients with greater instability, though such evidence is currently lacking. While the absence of a statistically significant difference in postoperative complications between IF and UIF groups in our study is encouraging, it should be viewed with caution due to the limited patient sample size, which may underrepresent rarer but serious adverse events. This emphasizes the importance of weighing potential gains in fusion stability with IF against the potential for increased surgical complexity and currently unclear associated risks in the LS population. Furthermore, factors such as bone quality, and overall spinal alignment can influence the decision. For example, patients with osteoporotic spines may experience suboptimal outcomes with IF due to poor screw purchase and reduced cortical density, potentially leading to screw loosening and necessitating reoperation.^33^, ^34^, ^35^ This further highlights the need for considering individual patient characteristics, ranging from but not limited to LS severity, overall bone quality, and preexisting comorbid burden when deciding to proceed with IF versus UIF.

Prior studies have examined the nuanced differences in outcomes between IF and UIF, including Hirase et al.'s (2022) systematic review, which highlighted the clinical implications of higher fusion rates in IF but was limited by its inclusion of predominantly observational studies.^12^ They found a significantly higher rate of fusion in IF compared to UIF, with fusion rates of 87.6 % and 77.1 %, respectively, similar to the trends in our study.^12^ However, Hirase et al. (2022) did not observe a difference in functional outcomes (e.g., ODI scores) between IF and UIF, which mirrors the findings of Wu et al. (2024) and Abdu et al. (2018), where both techniques yielded similar patient-reported outcomes despite the higher fusion rates in IF.^5^^,^^32^ A key distinction between our findings and those of Hirase et al. (2022) lies in their reliance on predominantly observational studies, which are prone to confounding biases that may skew outcomes. By focusing exclusively on RCTs, our review provides more robust evidence, allowing stronger conclusions about the equivalency of functional outcomes between IF and UIF. This higher-quality evidence also likely underpins our more definitive findings on reoperation rates and complications, areas less thoroughly examined by Hirase et al. (2022).

To address the limited number of trials, TSA was conducted for all primary outcomes, revealing that the cumulative Z-curve did not cross the required information size boundary for any outcome except fusion, which surpassed the significance boundary in favor of instrumentation. In contrast, reoperation and complication outcomes were fragile, with statistical significance sensitive to changes in just one patient's outcome. This underscores the inconclusiveness of current evidence, indicating that additional data—up to each outcome's required information size (range: 37–827)—is necessary to determine whether IF or UIF yields superior fusion or differential postoperative reoperation and complications, contributing to ongoing discussions about the safety and efficacy of IF versus UIF. Thus, surgeons carefully balance these findings against the individualized clinical benefits and risks for each patient. For instance, patients with fewer comorbidities but a greater need for structural stability may be more suitable candidates for IF, while those at higher surgical risk, with less pronounced instability or spondylolisthesis, might achieve adequate outcomes with UIF alone.

This study has several strengths. It provides a thorough meta-analysis of randomized trials comparing IF and UIF for LS, offering critical insights into postoperative outcomes such as fusion rates, reoperation incidence, and incidence of complications. The inclusion of TSA and FI also enhances the robustness of the conclusions, adding valuable quantitative tools to assess the sufficiency of existing evidence and help guide future research. By synthesizing data from multiple high-quality RCTs, this review reduces the risk of bias typically present in nonrandomized studies, thereby offering a stronger foundation for clinical recommendations. However, several limitations must be considered. While we aimed to focus on high-quality evidence, the heterogeneity in surgical techniques can introduce variability that could influence the outcomes. Additionally, the lack of blinding in these surgical trials increases the risk of performance and detection bias, which may affect the reported results, particularly for subjective outcomes like pain and disability scores. Another limitation is that our study was unable to comprehensively evaluate long term outcomes beyond the standard postoperative period (e.g. past 10 years) to examine fusion durability and the development of adjacent segment disease. This is particularly relevant in LS, where complications may arise years after the initial surgery. Furthermore, the TSA findings indicate that the current body of evidence is underpowered, suggesting that additional large-scale RCTs are needed to confirm or refute our conclusions definitively. Future research should focus on conducting larger RCTs with longer follow-up durations to assess both the short-term and long-term outcomes of IF and UIF in diverse patient populations.

## 5 Conclusion

This systematic review and meta-analysis provides a robust evaluation of IF versus UIF for isthmic and degenerative grade 1 and grade 2 LS. Although IF achieved statistically significant higher fusion rates than UIF, this benefit did not correlate with statistically significant reductions in reoperation or complication rates. Qualitative data suggest that IF may be associated with increased operative time and blood loss, though further investigation is warranted. TSA and FI analyses underscore the current limitations in available evidence, reinforcing the need for larger, high-quality RCTs to draw definitive conclusions. These findings ultimately highlight the importance of cautious, individualized surgical planning, with considerations for instability severity and comorbidities guiding the decision between IF and UIF. Future large, randomized trials should emphasize long-term outcomes, including fusion durability and adjacent segment disease, to provide more comprehensive guidance for optimizing patient care in lumbar spondylolisthesis management.

## CRediT authorship contribution statement

**Omkar S. Anaspure:** Conceptualization, Methodology, Formal analysis, Writing, Critial Revision. **Pierce Davis:** Methodology, Writing, Critial Revision. **Anthony N. Baumann:** Methodology, Critial Revision. **Meredith Kossoff:** Formal analysis, Critial Revision. **Gordon Preston:** Critial Revision. **Keegan T. Conry:** Critial Revision. **Jacob C. Hoffmann:** Conceptualization, Critial Revision.

## Informed consent statement

Informed consent was not required to gather the data to publish this paper.

## Institutional review board statement

Not applicable.

## Funding information

No funding was received for this study.

## Declaration of competing interest

The authors have no conflicts of interest to disclose.
